# Polystyrene Microplastic-Induced Cellular Alterations and Their Effects on Viral Entry (RSV, HCoV-OC43, EV-A71) and Viral Persistence

**DOI:** 10.1021/envhealth.5c00613

**Published:** 2026-02-05

**Authors:** Nattamon Niyomdecha, Pornprapa Srimorkun, Jarunee Prasertsopon, Kittisak Suanpan

**Affiliations:** † Department of Medical Technology, Faculty of Allied Health Sciences, 37698 Thammasat University, Rangsit Campus, Pathum Thani 12120, Thailand; ‡ Graduate Program in Biomedical Sciences, Faculty of Allied Health Sciences, 65100 Thammasat University, Pathum Thani 12120, Thailand; § Center for Research Innovation and Biomedical Informatics, Faculty of Medical Technology, 26685 Mahidol University, Nakhon Pathom 73170, Thailand; ∥ Department of Pediatrics, Faculty of Medicine Ramathibodi Hospital, 26685 Mahidol University, Bangkok 10400, Thailand

**Keywords:** environmental microplastics, environmental virology, host−virus interactions, viral persistence, low-temperature stability

## Abstract

Microplastics (MPs) are emerging environmental pollutants with biological impacts extending beyond cytotoxicity, yet their interactions with viruses remain poorly understood. This study systematically investigated the cellular and virological effects of polystyrene microplastics (PS-MPs) using three medically relevant viruses—human coronavirus OC43 (HCoV-OC43), respiratory syncytial virus (RSV), and Enterovirus A71 (EV-A71)—across multiple human cell lines under identical experimental conditions. PS-MP exposure induced dose- and time-dependent cytotoxicity, G2/M cell-cycle arrest, and early apoptosis, including effects observed at subcytotoxic concentrations. Coexposure assays revealed increased detectable infection levels and enhanced cold-temperature persistence of enveloped viruses (HCoV-OC43 and RSV), whereas no comparable effect was observed for the nonenveloped EV-A71, suggesting preferential effects on viral stability rather than intrinsic infectivity. Under coexposure conditions, PS-MPs reduced type I interferon (IFN-α/β) expression, indicating impaired innate antiviral signaling, while modulation of IFITM3 expression varied depending on viral species and host cell context. Gene-expression analyses demonstrated virus-specific antiviral modulation with attenuated interferon-mediated antiviral priming during HCoV-OC43 infection and reduced IFITM3 expression in RSV-infected cells. Collectively, these findings indicate that PS-MPs act as environmental and biological cofactors that induce cellular stress, suppress antiviral immune responses, and promote viral stability under low-temperature conditions, with implications for viral persistence in contaminated ecosystems.

## Graphical abstract

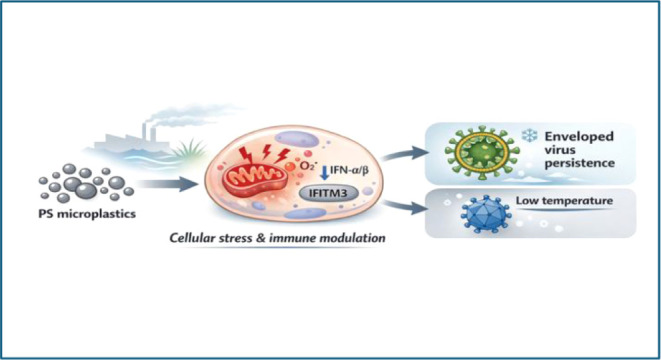

## Introduction

Plastics are inexpensive, lightweight, and widely manufactured materials, with annual global production now exceeding 400 million metric tons; growth is driven largely by single-use applications and low recycling rates, resulting in massive plastic waste accumulation.^1^ Owing to their resistance to degradation, bulk plastics fragment slowly via photodegradation, oxidation, hydrolysis, and mechanical abrasion to form microplastics (MPs: ∼100 nm–5 mm) and nanoplastics (NPs: <100 nm).^2^ MPs have become a major global environmental concern because of their widespread distribution and potential risks to both the environment and human health. These persistent particles are ubiquitous in aquatic environments, the atmosphere, and indoor dust, and human exposure primarily occurs through ingestion and inhalation, with possible uptake via dermal contact.^3^

Human biomonitoring now confirms MPs in multiple tissues and fluids, including blood, feces, sputum, lung tissue, and placenta.^4–6^ Experimental and review evidence link MP/NP exposure to oxidative stress, inflammatory signaling, epithelial barrier disruption, and metabolic disturbance across diverse mammalian cell models.^1,7^ These responses are characterized by the upregulation of proinflammatory mediators, such as IL-6 and IL-8, and by activation of stress pathways, which can plausibly modulate antiviral defenses at mucosal surfaces.^1^

Beyond direct toxicity, MPs may act as fomites and ecological vectors that adsorb and transport microbes. For viruses, three nonexclusive mechanisms are increasingly supported: (i) surface adsorption and shielding, which can prolong environmental infectivity; (ii) plastisphere biofilm effects, which stabilize virions on colonized plastics; and (iii) cell-entry facilitation when MP–virus complexes contact host cells.^8–10^ In one freshwater microcosm study, the nonenveloped rotavirus SA11 maintained infectivity for up to 3 days primarily through direct attachment to MP beads, whereas the enveloped surrogate Phi6 required colonization by biofilms on MPs for recovery, suggesting potential differences in the mechanisms of persistence between these nonenveloped and enveloped viruses.^8^ In human-cell systems, polystyrene MPs (PS-MPs) bound a severe acute respiratory syndrome coronavirus 2 (SARS-CoV-2) pseudovirus and increased infection, with confirmation using authentic SARS-CoV-2.^10^ Mechanistically, MPs can also amplify influenza A virus infection in A549 cells by suppressing innate antiviral signaling—downregulating RIG-I, TBK1 phosphorylation, IRF3 activation, and IFN-β, alongside IFITM3 reduction—thus tipping the cellular response toward higher viral yields.^9^ Together, these findings suggest that MP exposure may enhance viral persistence and transport in the environment and increase host susceptibility by impairing early antiviral defenses, providing a biologically plausible framework for MP–virus interactions relevant to respiratory and enteric infections.

This study aims to investigate the biological effects of PS-MPs on host cells and determine whether MP exposure modulates viral infection outcomes. Unlike the study by Wang et al.,^9^ which targeted a single enveloped virus (influenza A virus), this study directly compares two enveloped viruses—human coronavirus OC43 (HCoV-OC43) and respiratory syncytial virus (RSV)—and one nonenveloped virus, Enterovirus A71 (EV-A71), to clarify envelope-dependent differences in the effects of PS-MPs. This design enables a direct assessment of whether the viral envelope status influences MP-associated effects. Importantly, the study integrates previral cellular effects—including cytotoxicity, cell-cycle perturbation, and apoptosis—with downstream outcomes such as viral persistence, detectable infection levels, and host antiviral responses. Accordingly, a multilevel experimental strategy was employed, progressing from cellular stress responses to virus–MP coexposure assays and molecular analyses of innate immune and entry-related pathways. This integrated approach provides a comprehensive framework for understanding how microplastic pollution may modulate virus–host interactions and contribute to viral persistence and transmission risks in contaminated environments.

## Materials and Methods

### Microplastics

Synthetic PS-MPs (100 nm mean diameter, ≤10 nm standard deviation) were obtained from Sigma-Aldrich (Cat. No. 43302). The particles were supplied as an aqueous suspension at 10% w/v with a density of 1.05 g/cm^3^. Further product characterization details provided by the manufacturer are presented in Table S1. Working concentrations of 25, 50, 100, 200, 500, and 1000 ng/μL were prepared by serial dilution of the stock suspension using sterile ultrapure distilled water. All prepared suspensions were stored at 2–8 °C until use.

### Cell Lines and Viruses

HCT-8 (human ileocecal adenocarcinoma; ATCC CCL-244), RD (human rhabdomyosarcoma), HEp-2 (human laryngeal epithelioma), and Vero (African green monkey kidney) cell lines were selected to represent distinct anatomical sites and exposure routes relevant to viral infection and PS-MP entry into the human body, including the gastrointestinal and respiratory tracts, which are major portals for both virus transmission and PS-MP uptake. In addition, these cell lines support infection by viruses with different structural properties (enveloped and nonenveloped), enabling comparative evaluation of PS-MP–virus–host interactions across biologically relevant *in vitro* models.

HCT-8 cells were cultured in RPMI-1640 medium supplemented with 10% horse serum and antibiotics. RD, HEp-2, and Vero cells were maintained in their respective media—DMEM for RD and MEM for HEp-2 and Vero—each supplemented with 10% fetal bovine serum (FBS) and antibiotics. All cell cultures were incubated at 37 °C in a humidified atmosphere with 5% CO_2_.

HCoV-OC43 (strain VR-1558) was propagated in susceptible HCT-8 and RD cells, while RD cells were exclusively used for plaque titration assays due to their consistent plaque formation and suitability for quantitative viral enumeration. RSV-Long subgroup A (RSV-A, VR-26TM) and EV-A71 (strain SilCRC10/TH/2011) were propagated in HEp-2 and Vero cells, respectively. Although some of these cell lines do not represent the primary *in vivo* target cells of infection, they express functional host receptors and intracellular machinery that permit productive viral entry and replication, making them well-established and widely accepted *in vitro* models for mechanistic viral infection studies. Virus stocks were harvested, clarified by centrifugation to remove cellular debris, and stored in aliquots at −80 °C until use.

### Cell Viability Analysis

All cell lines were exposed to PS-MPs at concentrations of 0, 25, 50, 100, 200, 500, and 1000 ng/μL for 1, 2, 4, and 6 days in growth medium supplemented with 2% FBS. Cell viability was assessed using the MTT assay, which measures the reduction of 3-(4,5-dimethylthiazol-2-yl)-2,5-diphenyltetrazolium bromide to insoluble formazan crystals by metabolically active cells. After incubation with MTT, the resulting formazan precipitates were solubilized in DMSO, and the absorbance was measured at 570 nm. Cells treated with 0 ng/μL PS-MPs were used as the mock control, with viability normalized to 100%. The percentage of viable cells in PS-MP-treated groups was calculated relative to the control based on optical density (OD) values. The experiments were performed in technical duplicates.

### Cell Cycle Analysis and Apoptosis Detection Assay

RD and Vero cells were seeded in T25 flasks at a density of 2.5 × 10^6^ cells in growth medium supplemented with 10% FBS. Cells were either left unexposed (mock control) or exposed to PS-MPs at a concentration of 200 ng/μL for 1, 3, and 5 days. Following exposure, cells were subjected to cell cycle and apoptosis analyses.

Cell cycle analysis was performed using Propidium Iodide (PI) staining. RD and Vero cells were harvested and washed with ice-cold 1× DPBS. The cell concentration was adjusted to 1.0 × 10^6^ cells/mL. The cells were fixed in cold 70% ethanol for at least 30 min by adding ethanol dropwise to the cell pellet while vortexing. Ethanol was subsequently removed from the cells by centrifugation at 900*g* for 5 min and washed twice with ice-cold 1× DPBS. To eliminate RNA, RNase A (250 ng/μL, iNtRON Biotechnology, South Korea) was added at a final concentration of 5 μg per 200 μL of cell suspension. After that, 5 μL of PI (500 ng/μL, BioLegend, USA) was added to each sample. The stained cells were analyzed by using flow cytometry to determine the proportion of cells in the three interphase stages of the cell cycle: G1, S, and G2/M phases.

Apoptosis was evaluated using Annexin V-FITC and 7-AAD dual staining. RD and Vero cells were harvested, washed three times with 1× DPBS (Pan Biotech, Germany), and resuspended at 0.5 × 10^6^–1.0 × 10^6^ cells/200 μL. Each cell suspension was incubated with 2 μL of Annexin V-FITC conjugate (90 ng/μL; BioLegend, USA) and 2 μL of 7-AAD (50 ng/μL; BioLegend, USA) for 20 min at room temperature in the dark. Samples were analyzed using a BD FACSLyric flow cytometer (USA), and data were processed with FlowJo software, version 10.0 (BD, USA), to distinguish viable, early apoptotic, late apoptotic, and necrotic cell populations.

The experiments were performed in two independent biological replicates, with each analyzed in duplicate. For flow cytometric analysis, a minimum of 10,000 events was acquired per sample.

### Coexposure between Microplastics and Viruses

#### Viral Infectivity Assay

Cells were seeded in 24-well plates at a density of 2.2 × 10^5^–2.7 × 10^5^ cells/well in growth medium supplemented with 10% FBS. After overnight incubation to reach 80%–90% confluence, the medium was replaced with virus inoculum prepared in growth medium containing 2% FBS at a multiplicity of infection (MOI) of 0.005.

Coexposure experiments were performed by infecting HCT-8 and RD cells with HCoV-OC43, HEp-2 cells with RSV, and Vero cells with EV-A71, in the presence of PS-MPs at final concentrations of 0, 50, 100, or 200 ng/μL. Infected cultures were incubated under standard conditions for virus-specific incubation periods corresponding to the time required for productive infection, namely 4 days for HCoV-OC43 in RD cells, 7 days for HCoV-OC43 in HCT-8 cells, and 3 days for both RSV and EV-A71. Viral titers were subsequently quantified by a plaque assay using the respective host cell lines. The experiments were performed twice as independent biological replicates.

#### Viral Persistence Assay

An identical viral inoculum of 10^4^ PFU/100 μL was prepared for all viruses and mixed with PS-MPs at final concentrations of 0, 50, and 200 ng/μL. The mixtures were divided into two sets and stored at either 4 or 30 °C (defined as room temperature for tropical environmental conditions) for 3, 5, and 7 days. At each time point, residual viral infectivity was quantified by plaque assay. The percentage of viral survival was calculated relative to the initial inoculum titer. The experiments were performed in technical duplicates.

### Plaque Assay

Viral titers were quantified by using a standard plaque assay. Cells were seeded in 24-well plates at a density of 2.2 × 10^5^–2.7 × 10^5^ cells/well and incubated overnight to form confluent monolayers. Subsequently, 100 μL of 10-fold serially diluted virus samples from the culture supernatant were added to each well and allowed to adsorb for 1 h at 37 °C in a 5% CO_2_ atmosphere. After adsorption, the inoculum was removed, and cells were overlaid with 1.2%–1.56% Avicel RC-591 prepared in growth medium containing 2% FBS. The plates were then incubated under standard conditions for virus-specific durations—4 days for HCoV-OC43, 7 days for RSV, and 3 days for EV-A71. Following incubation, monolayers were fixed with 10% formalin in PBS for 2 h and stained with 1% crystal violet in 20% ethanol. Visible plaques were enumerated, and viral titers were calculated as plaque-forming units per milliliter (PFU/mL).

### mRNA Gene Expression Analysis

Following the coexposure experiment for viral infectivity assessment, total RNA was extracted from cells after 24 h of incubation using TRIzol reagent (Invitrogen, USA). Gene expression of interferon-alpha (IFN-α), interferon-beta (IFN-β), interferon-induced transmembrane protein 3 (IFITM3), and the housekeeping gene GAPDH was quantified by reverse transcription quantitative PCR (RT-qPCR). Primer sequences are listed in Table 1.

**Table 1:** Primer Sequences Used for Host Anti-Viral Gene Expression Analysis

Target genes	Sequences (5′-3′)	References
IFN-α	F: GCCTCGCCCTTTGCTTTACT	Wang et al.^9^
	R: CTGTGGGTCTCAGGGAGATCA	
IFN-ß	F: ATGACCAACAAGTGTCTCCTCC	Wang et al.^9^
	R: GGAATCCAAGCAAGTTGTAGCTC	
IFITM3	F: CCCAGGGACCTCTCTCTAATCA	Wang et al.^9^
	R: GCTTGAGGGTTTGCTACAACATG	
Human-GAPDH	F: GTCTCCTCTGACTTCAACAGCG	Tan et al.^11^
	R: ACCACCCTGTTGCTGTAGCCAA	
Vero-GAPDH	F: CAGCCTCAAGATCGTCAGCA	Shiimura et al.^12^
	R: TCTTCTGGGTGGCAGTGATG	

Reactions were prepared using the Luna Universal One-Step RT-qPCR Kit (New England Biolabs, USA) in a total volume of 20 μL, containing 1× One-Step Reaction Mix, 1× WarmStart RT Enzyme Mix, 200 nmol/L of each primer, 2 μL of total RNA template (corresponding to 100 ng of RNA), and nuclease-free water. Amplification was performed on a CFX-96 Real-Time PCR Detection System (Bio-Rad, USA) under the following cycling conditions: reverse transcription at 55 °C for 10 min, initial denaturation at 95 °C for 1 min, followed by 40 cycles of 95 °C for 10 s and 60 °C for 30 s, and a final melt-curve analysis to verify amplicon specificity.

Quantification cycle (*Cq*) values were obtained directly from each reaction, and relative gene expression was determined using the 2^–ΔΔ*Ct*^ method with GAPDH serving as the internal reference gene. Expression levels were normalized to GAPDH and presented relative to the mock control group. The experiments were performed twice as independent biological replicates.

### Replication, Statistical Analysis, and Data Visualization

Replication strategies were selected according to assay type, with sample sizes (*n* = 2 or 4) specified in the corresponding method descriptions. Unpaired *t*-tests were used for comparisons between individual treatment groups and their respective controls or for predefined pairwise comparisons, as appropriate. Two-way analysis of variance (ANOVA) with Bonferroni correction was applied when multiple independent variables were analyzed simultaneously. Statistical significance was defined as a *p*-value < 0.05. Statistical analyses were performed using the online GraphPad QuickCalcs tool (https://www.graphpad.com/quickcalcs/) or GraphPad Prism version 10 (GraphPad Software, USA). Graphs were generated using GraphPad Prism version 10.

### Biosafety Approval

All procedures involving biological risk group viruses were approved by the Thammasat University Institutional Biosafety Committee (Approval No. 044/2567). Appropriate biosafety level (BSL) laboratory facilities and practices were implemented in accordance with the assigned biological risk classification for each virus.

## Results

### Biological Effects of Microplastics

As illustrated in Figure 1, exposure to PS-MPs-induced cytotoxicity in a time- and dose-dependent manner across all examined cell lines. A significant reduction in cell viability was first evident on day 2 in HCT-8 and RD cells exposed to PS-MP concentrations ≥500 ng/μL, with cytotoxicity progressively increasing over prolonged exposure. In contrast, HEp-2 and Vero cells demonstrated a comparatively delayed response, showing marked cellular damage only by day 4 at similarly high PS-MP concentrations (≥500 ng/μL). These findings suggest that the sensitivity to PS-MP-induced cytotoxicity may vary among cell types, possibly reflecting differences in cellular uptake efficiency, surface receptor interactions, or intrinsic defense mechanisms against particle-induced stress.

**Figure 1:**
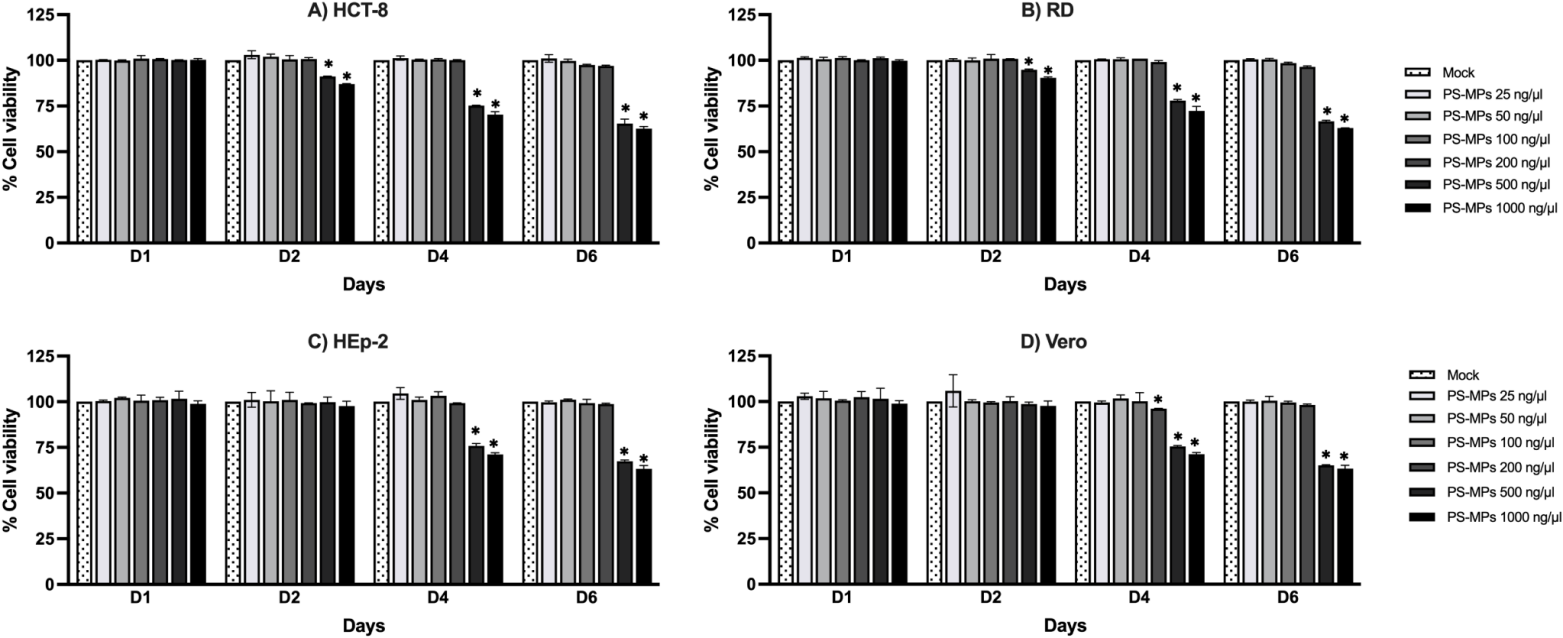
Time- and dose-dependent cytotoxicity of PS-MPs in different cell lines. HCT-8, RD, HEp-2, and Vero cells were exposed to PS-MPs (0–1,000 ng/μL) for 1–6 days. Cell viability was measured using a colorimetric (MTT) assay and expressed as a percentage relative to the mock control (0 ng/μL PS-MPs) for the corresponding day. Data are presented as mean ± SD from technical duplicates (*n* = 2). Statistical analysis was performed using an unpaired *t*-test comparing each PS-MP-treated group with the mock control at the same time point. Asterisks indicate statistically significant differences (*p < 0.05).

Although exposure to 200 ng/μL of PS-MPs did not cause significant cytotoxicity in the viability assay, this concentration was selected for subsequent mechanistic studies to examine subtle or early cellular responses. Such subcytotoxic exposure levels are often sufficient to trigger stress signaling, cell-cycle modulation, or apoptosis without inducing overt cell death, thereby allowing detection of early biological alterations preceding toxicity.

To further investigate the cellular response to PS-MPs, RD and Vero cells were treated with 200 ng/μL PS-MPs for 24, 72, and 120 h, and cell-cycle distribution was analyzed by flow cytometric measurement of nuclear DNA content. In RD cells, PS exposure significantly increased the proportion of cells in the G2/M phase within 24 h compared with the mock controls, accompanied by a corresponding decrease in the G1 phase population (Figure 2A). Similarly, Vero cells exhibited significant accumulation in the G2/M phase at both 24 and 72 h (Figure 2B). These findings indicate that PS-MPs exposure induces G2/M phase arrest in both cell lines as early as 24 h post-treatment.

**Figure 2:**
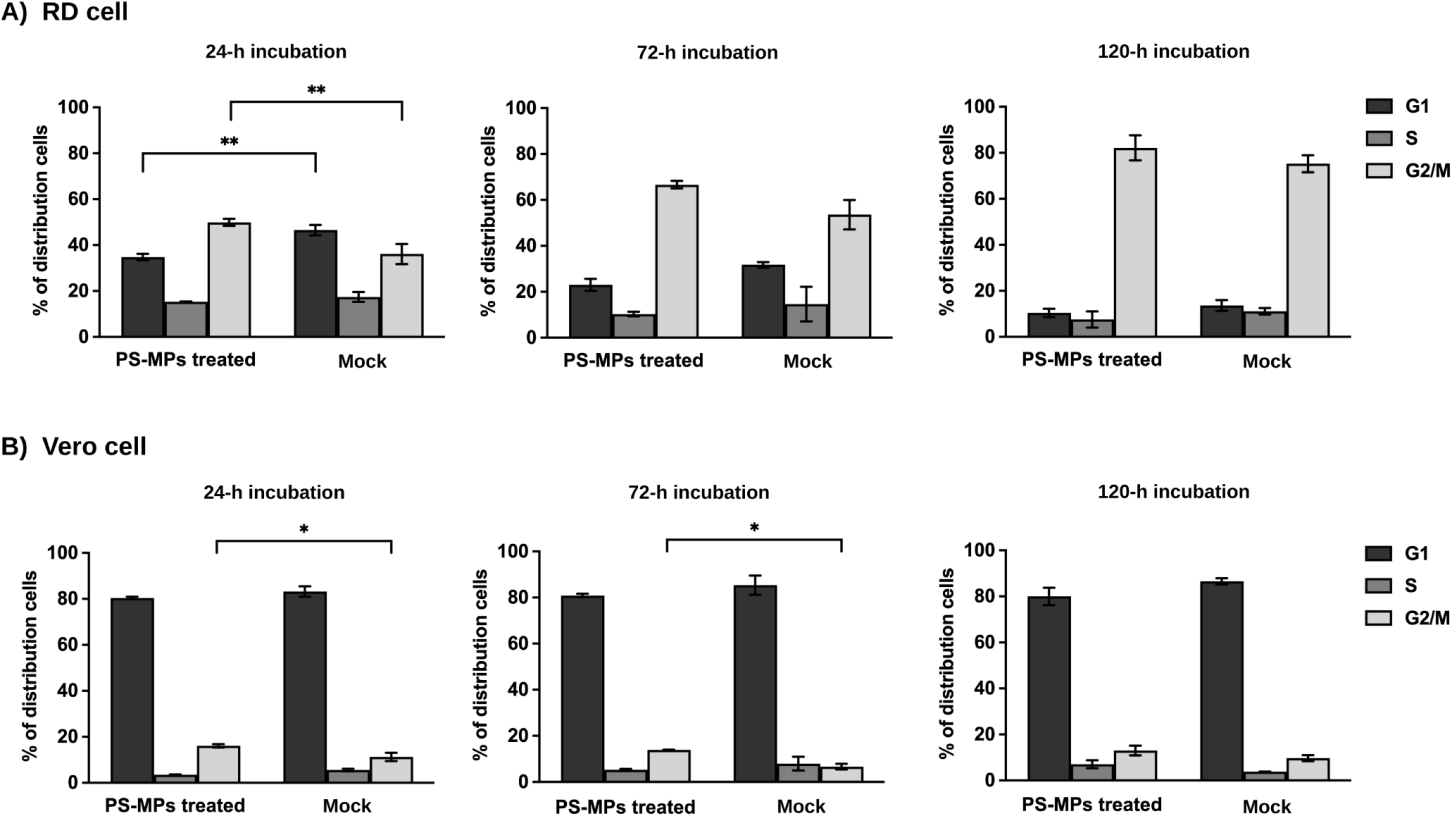
Cell-cycle distributions following PS-MP exposure. RD and Vero cells were exposed to 200 ng/μL PS-MPs for 24, 72, and 120 h. Cell-cycle distribution was analyzed by flow cytometry following DNA staining. Data are presented as the mean ± SD from two independent biological experiments performed in duplicate (*n* = 4). Statistical analysis was conducted using two-way ANOVA with Bonferroni correction to assess the effects of PS-MP exposure and incubation time. Asterisks indicate statistically significant differences compared with the corresponding mock control (*p < 0.05, **p < 0.01).

Following G2/M arrest, apoptosis was evaluated using Annexin V and 7-AAD staining and analyzed by flow cytometry (Figure 3). In Vero cells, PS-MPs exposure significantly increased the proportion of early apoptotic cells at all time points (24, 72, and 120 h), with a progressive rise over time. In contrast, RD cells showed no significant change in early apoptosis at 24 h, but marked increases were observed at 72 and 120 h. These results collectively demonstrate that treatment with 200 ng/μL PS-MPs triggers G2/M phase arrest followed by time-dependent apoptotic activation, particularly pronounced in Vero cells.

**Figure 3:**
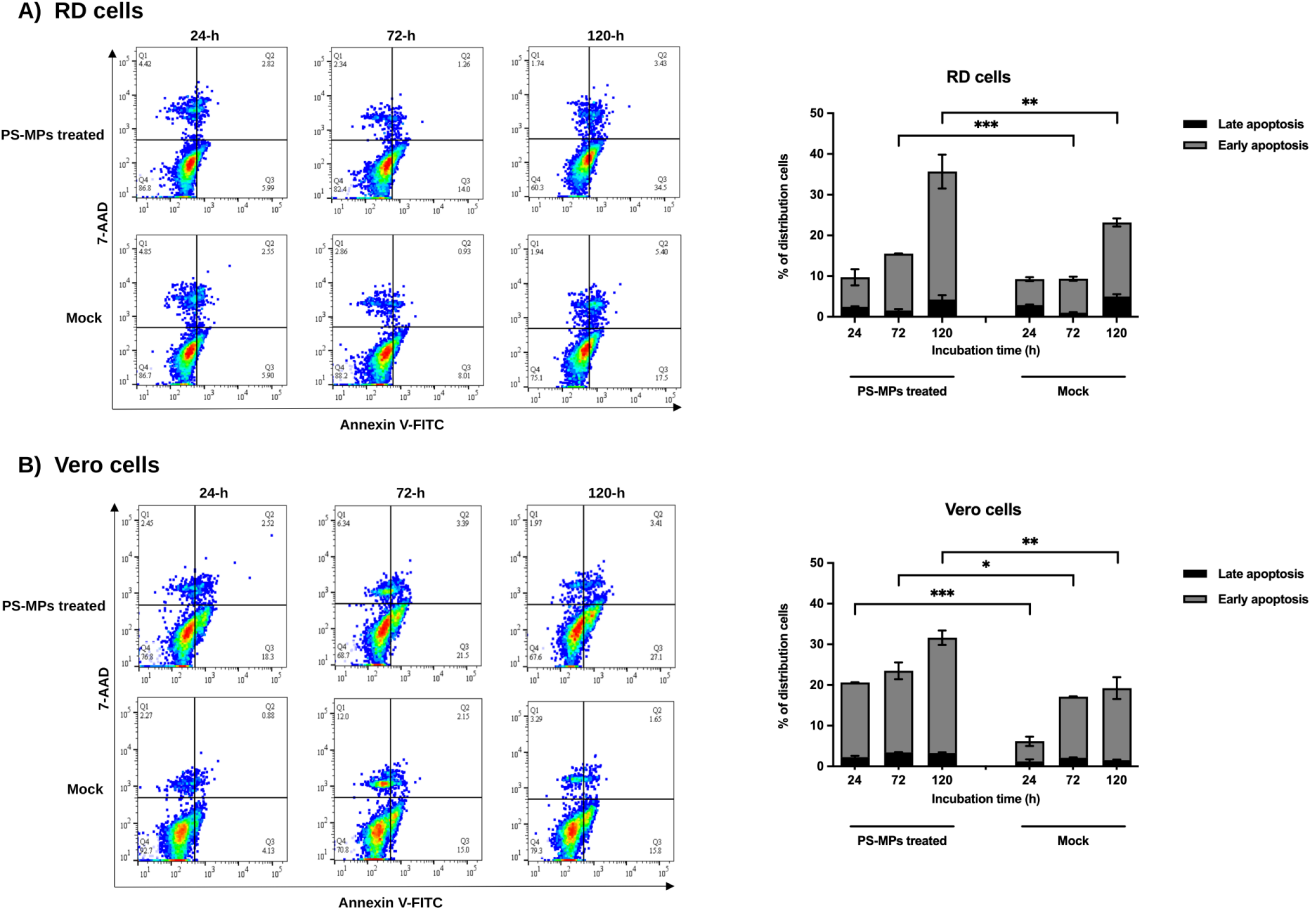
Apoptotic responses following PS-MP exposure. Apoptosis was assessed in RD and Vero cells after treatment with 200 ng/μL PS-MPs for 24, 72, and 120 h using Annexin V/7-AAD staining and flow cytometric analysis. Data are presented as mean ± SD from two independent biological experiments performed in duplicate (*n* = 4). Statistical analysis was conducted using two-way ANOVA with a Bonferroni correction to evaluate the effects of PS-MP exposure and incubation time. Asterisks indicate statistically significant differences compared with the corresponding mock control (*p < 0.05, **p < 0.01, ***p < 0.001).

Together, these findings suggest that even at subcytotoxic concentrations, PS-MPs can disrupt cell cycle progression and initiate apoptotic signaling, implying that early cellular stress responses precede measurable cytotoxicity and may contribute to long-term cellular dysfunction.

### Effects of Microplastics on Viral Infection and Persistence

To determine whether PS-MPs influence viral infectivity, HCoV-OC43, RSV, and EV-A71 were coincubated with PS-MPs at concentrations of 0, 50, 100, and 200 ng/μL. As shown in Figure 4, PS-MPs were associated with increased viral titers of HCoV-OC43 and RSV at specific concentrations in a nonlinear, dose-responsive manner. In HCoV-OC43, the enhancement was most evident in both HCT-8 and RD cell lines, whereas in RSV, the viral titer increased prominently at 200 ng/μL. In contrast, no significant difference was observed in EV-A71 infection at any PS-MP concentration compared to virus controls. These findings suggest that PS-MPs can facilitate viral entry or replication in certain enveloped viruses (HCoV-OC43 and RSV) but not in the nonenveloped virus (EV-A71), indicating that viral structural characteristics may modulate the interaction with microplastic particles.

**Figure 4:**
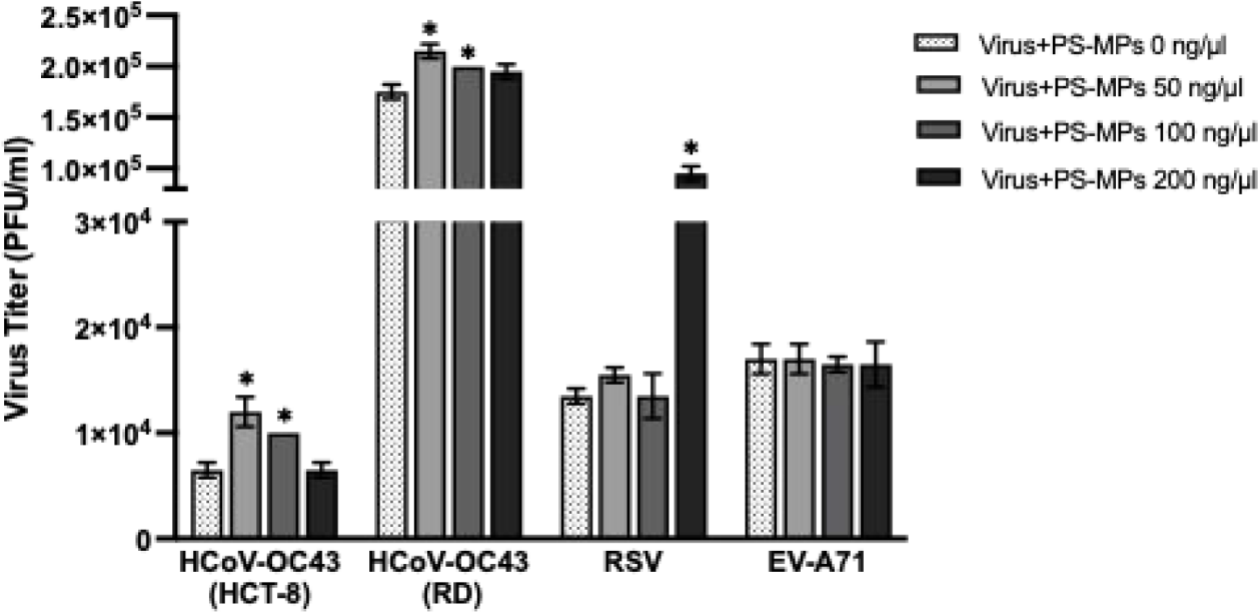
Effects of PS-MP on viral infection. HCoV-OC43, RSV, and EV-A71 were coincubated with PS-MPs at concentrations of 0, 50, 100, and 200 ng/μL. Viral titers in the culture supernatants were quantified by the plaque assay and expressed as PFU/mL. Data are presented as mean ± SD from two independent biological experiments (*n* = 2). Statistical analysis was performed using an unpaired *t*-test comparing each PS-MP–coexposured group with the corresponding virus control without PS-MP exposure (0 ng/μL PS-MPs). Asterisks indicate statistically significant differences (*p < 0.05).

To further assess whether PS-MPs influence viral stability, each virus was directly exposed to PS-MPs at 4 °C for various durations. As shown in Figure 5, PS-MPs markedly preserved the viral infectivity of HCoV-OC43 and RSV compared with nonexposed controls. For HCoV-OC43, the protective effect was evident across all tested days, while for RSV, it was significant on days 3 and 5. Conversely, EV-A71 showed no change in titer following PS-MP exposure, suggesting that PS-MPs did not confer stability to the nonenveloped virus. Temperature strongly influenced these outcomes: when the same experiments were conducted at room temperature (30 °C), none of the viruses—regardless of PS-MP exposure—remained detectable by day 3 (Figure S1). These results indicate that PS-MPs can enhance viral stability and persistence under cold conditions, particularly for enveloped viruses. Such interactions may stem from electrostatic or hydrophobic associations between the viral envelope and PS surfaces, which could shield virions from degradation and potentially prolong their environmental survival.

**Figure 5:**
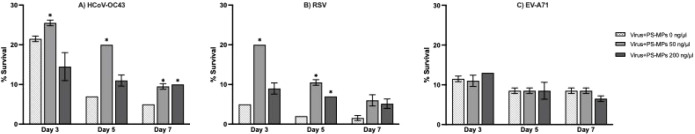
Effects of PS-MP on viral persistence. HCoV-OC43, RSV, and EV-A71 were incubated with PS-MPs at concentrations of 0, 50, and 200 ng/μL at 4 °C for the indicated durations, and residual viral infectivity was determined by plaque assay and expressed as a percentage of survival. Data are presented as mean ± SD from technical duplicates (*n* = 2). Statistical analysis was performed using an unpaired *t*-test comparing each PS-MP–coexposured group with the corresponding virus control without PS-MP exposure (0 ng/μL PS-MPs) at the same time point. Asterisks indicate statistically significant differences (*p < 0.05).

Together, these results show that PS-MPs were associated with increased viral titers of enveloped viruses and prolonged viral detectability under cold-temperature conditions, whereas no comparable effect was observed for the nonenveloped virus.

### Preliminary Evaluation of the Underlying Mechanism

Previous studies have reported that PS-MPs may promote viral infection by interfering with host antiviral signaling pathways. Consistent with this concept, the present study investigated the expression of key antiviral genes—IFN-α, IFN-β, and IFITM3—to explore possible mechanisms underlying the enhanced infectivity observed in PS-MP–exposed cells.

As shown in Figure 6, exposure to PS-MPs significantly reduced the expression of IFN-α and IFN-β in RD cells and suppressed all three genes (IFN-α, IFN-β, and IFITM3) in HEp-2 cells compared with the mock controls. Notably, while HCoV-OC43 infection alone markedly upregulated type I interferon expression, coexposure with PS-MPs attenuated this virus-induced interferon activation, indicating that PS-MPs can dampen antiviral signaling even in the presence of a strong interferon-stimulating infection. In contrast, PS-MP exposure did not significantly affect IFITM3 expression during HCoV-OC43 infection in either single-treatment or coexposure conditions. Therefore, the interferon-facilitating effect of PS-MPs in HCoV-OC43 appears to be primarily associated with suppression of type I interferon signaling rather than IFITM3 modulation. Importantly, this observation should not be generalized across viruses, as PS-MP-associated suppression of IFITM3 was observed specifically in RSV-infected HEp-2 cells, indicating a virus- and cell-dependent effect.

**Figure 6:**
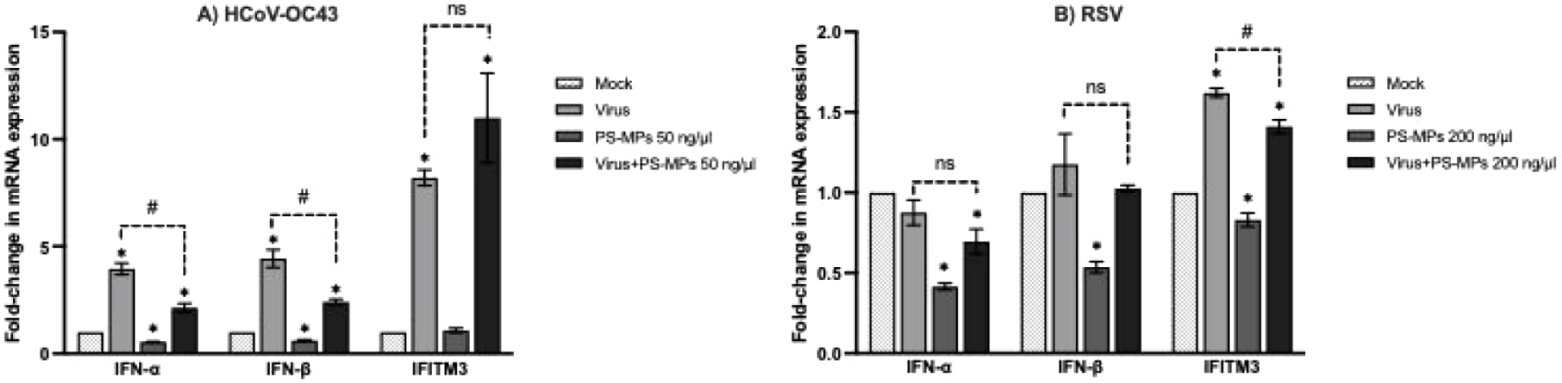
Effect of PS-MP on antiviral gene expression. Relative mRNA expression levels of IFN-α, IFN-β, and IFITM3 were analyzed by quantitative RT-PCR in (A) HCoV-OC43-infected RD cells and (B) RSV-infected HEp-2 cells following exposure to PS-MPs. Gene expression levels were normalized to GAPDH, and the mock control was set to a relative value of 1. Data are presented as mean ± SD from two independent biological experiments (*n* = 2). Statistical analysis was performed using unpaired *t*-tests. Asterisks indicate statistically significant differences compared with the mock control (*p < 0.05). Dashed brackets with corresponding significance labels denote direct statistical comparisons between the virus control without PS-MP exposure (0 ng/μL PS-MPs) and virus + PS-MP-treated groups (#*p* < 0.05; ns, not significant).

For RSV infection (Figure 6B), the virus alone did not significantly affect IFN-α or IFN-β expression compared with the mock control, and coexposure with PS-MPs did not produce additional suppression. However, PS-MP exposure alone or in combination with RSV markedly reduced IFITM3 expression, which may contribute to increased viral susceptibility by impairing this critical antiviral restriction factor.

Together, these results suggest that PS-MPs can compromise host innate immune responses through the selective suppression of type I interferon signaling and IFITM3 expression, thereby creating a cellular environment more permissive to viral infection and replication. This mechanistic evidence complements the observed increases in viral infectivity and persistence, highlighting the potential role of microplastics as cofactors that modulate antiviral defense pathways.

## Discussion

These findings address a critical gap in current knowledge regarding microplastic–virus interactions. Microplastics are known to accumulate locally at elevated levels in specific microenvironments, such as biofilms, sediments, wastewater systems, and cell–particle interfaces.^13,14^ Although the PS-MP concentrations used in this study (25–1,000 ng/μL) exceed most reported average environmental levels were intentionally selected as a mechanistic screening range rather than to replicate bulk exposure. Importantly, measurable effects on cellular stress responses and antiviral signaling were observed at subcytotoxic concentrations, indicating high sensitivity of host–virus interactions to PS-MP exposure. Consistent with prior *in vitro* microplastic studies, comparable concentration ranges have been widely employed to interrogate early cellular and immunological perturbations under controlled experimental conditions.^15,16^ Accordingly, a comparison between the concentration range used in this study and reported environmental and human exposure levels is provided in Table S2, highlighting the distinction between bulk environmental measurements and localized microplastic accumulation at biological interfaces.

Consistent with previous studies showing that PS-MPs generate oxidative stress and mitochondrial dysfunction,^17,18^ our results revealed time- and dose-dependent cytotoxicity in all tested cell lines, accompanied by G2/M-phase arrest and early apoptotic activation even at subcytotoxic concentrations. These findings suggest that PS-MPs can trigger stress-response signaling through ROS accumulation and p53/p21 pathway activation prior to overt cytotoxicity. Remarkably, despite these stress responses, PS-MP exposure led to a significant reduction in type I interferon (IFN-α and IFN-β) expression, indicating that mitochondrial or endoplasmic reticulum stress induced by microplastics may disrupt RIG-I/MAVS–IRF3 signaling and dampen innate antiviral activation.^9^ This suppression aligns with previous reports that PS-MPs can downregulate IFN-β and IFITM3 in influenza-infected A549 cells,^9^ reinforcing the concept that microplastics interfere with host antiviral transcriptional responses.

In the viral infectivity assays, PS-MP coexposure differentially affected viral outcomes, with increased detectable infection levels observed for the enveloped viruses HCoV-OC43 and RSV, but not for the nonenveloped EV-A71. This pattern suggests virus-type-dependent effects and, based on existing literature, may reflect multiple physicochemical interactions, including hydrophobic compatibility between polystyrene surfaces and viral lipid envelopes, electrostatic interactions between viral envelope glycoproteins and the zeta potential of PS-MPs, and potential PS-MP-induced changes in host cell membrane fluidity that could facilitate membrane fusion of enveloped viruses. In contrast, nonenveloped viruses, whose rigid protein capsids lack lipid-mediated hydrophobic interfaces, exhibit weaker affinity for PS-MPs and are therefore less affected.^19,20^ Using model viral surrogates, Lu et al.^19^ experimentally demonstrated that adsorption efficiency increased with particle hydrophobicity and depended strongly on zeta potential and ionic strength, indicating that van der Waals and electrostatic forces jointly govern viral attachment to polystyrene surfaces. Consistent with this mechanism, polystyrene microplastics have been shown to directly interact with SARS-CoV-2 particles and facilitate host cell infection, supporting the role of envelope-associated glycoprotein-mediated binding in coronavirus–microplastic interactions.^10^ Similar facilitative effects have also been reported for other enveloped respiratory viruses, including influenza A virus, in which PS-MPs enhanced viral infection through combined physicochemical interactions and modulation of host antiviral responses.^9^ These findings collectively support the hypothesis that enveloped viruses, which possess lipid bilayers rich in hydrophobic domains, are more likely to adsorb onto PS-MPs than nonenveloped viruses that lack such interfaces.

However, not all studies showed the same pattern. Moresco et al.^8^ reported that in aquatic environments, the nonenveloped rotavirus SA11 retained infectivity longer than the enveloped Phi6 when attached to pristine microplastic beads. The discrepancy likely reflects environmental context and particle conditions—Phi6, being lipid-enveloped, is more sensitive to desiccation, oxidation, and temperature, whereas SA11’s protein capsid confers greater intrinsic stability. In contrast, under the controlled cellular and low-temperature conditions of this study, PS-MPs may act as protective carriers for enveloped viruses by reducing envelope degradation and preserving hydration at the particle interface.

The distinctive physicochemical properties of microplastics—including their large specific surface area, pronounced hydrophobicity, and high adsorption capacity—thus create favorable microenvironments that can mediate virus–particle interactions and potentially promote viral persistence and transmission in contaminated ecosystems.

Consistent with this interpretation, PS-MPs markedly preserved the infectivity of HCoV-OC43 and RSV at 4 °C, while all viruses, including EV-A71, lost viability at room temperature. Numerous reports support that low temperatures stabilize viral envelopes and extend infectivity by multiple mechanisms: slowing lipid oxidation and protein denaturation of envelope components, maintaining the prefusion conformation of viral fusion proteins (e.g., RSV F protein), and reducing the degradation rate of viral RNA polymerases.^21,22^ Such temperature-dependent effects have also been observed for SARS-CoV-2 and coronaviruses, which remain viable for days at 4 °C but rapidly lose infectivity under ambient conditions.^21,22^ Together, these findings suggest that both the viral envelope composition and environmental factors—particularly temperature and hydration—govern how microplastics influence viral adsorption and persistence.

Mechanistically, growing evidence indicates that microplastics can interfere with the host immune homeostasis. MPs have been shown to alter immune-related enzyme activities, disrupt mitochondrial function, and dysregulate the expression of key immune-regulatory genes, thereby impairing immune competence and increasing susceptibility to infection and mortality in exposed organisms.^23–25^ In line with these findings, this study provides molecular evidence that PS-MPs modulate antiviral responses differently across viral species. Recent studies have shown that, unlike many other coronaviruses, HCoV-OC43 can exploit IFITM3 to enhance its own replication, as IFITM3 facilitates viral entry and fusion within endosomal compartments rather than restricting it.^26,27^ In this study, PS-MPs suppressed IFN-α and IFN-β transcription without significantly altering IFITM3 levels, suggesting that PS-MP exposure preferentially weakens interferon-mediated antiviral priming while preserving IFITM3-dependent entry pathways that could be exploited by HCoV-OC43. This pattern is consistent with stress-mediated disruption of upstream innate immune signaling rather than direct interference with viral entry factors and supports the notion that IFITM3 plays a proviral role in HCoV-OC43 infection, in contrast to its well-established antiviral function against other enveloped viruses. In contrast, RSV inherently suppresses IFN-α/β signaling through its nonstructural proteins NS1 and NS2,^28^ which likely accounts for the minimal interferon induction observed during coexposure. Under these conditions, PS-MP-induced cellular stress may instead target membrane-associated restriction factors. Indeed, PS-MPs markedly reduced IFITM3 expression in RSV-infected cells, potentially relieving membrane-fusion constraints and promoting viral entry. Together, these findings indicate that PS-MPs act on distinct antiviral checkpoints depending on the virus—primarily interferon suppression in HCoV-OC43 and IFITM3 downregulation in RSV—thereby creating a cellular environment more permissive to infection.

The apparent coexistence of immune suppression with cell-cycle arrest and apoptosis may initially appear paradoxical but can be reconciled by the multifaceted stress responses elicited by PS-MPs. Oxidative stress and DNA damage activate checkpoint arrest and early apoptotic pathways, whereas chronic mitochondrial dysfunction has been shown to impair innate antiviral signaling by promoting MAVS degradation and inhibiting IRF3 phosphorylation.^29^ Although mitochondrial function was not directly assessed in this study, such stress-mediated disruption provides a plausible cellular context linking PS-MP exposure to weakened interferon signaling and virus-specific modulation of antiviral defenses. Consequently, PS-MPs may generate a cellular state characterized by metabolic distress coupled with reduced antiviral readiness, a condition that could favor viral replication or persistence rather than efficient clearance. This integrated framework highlights how general cellular stress induced by environmental microplastics can intersect with virus-specific immune strategies to exacerbate susceptibility to infection.

This study has several limitations. Mechanistic analyses were primarily conducted at the transcriptional level; therefore, protein-level validation and functional immune assays were not included, consistent with the screening-oriented design across multiple viruses and cell models. In this context, future protein-level validation should prioritize key antiviral factors identified in the present study, particularly IFITM3 protein expression in RSV-infected HEp-2 cells and IFN-β secretion quantified by ELISA. These targets represent critical downstream effectors of the observed transcriptional changes and provide functional confirmation of the PS-MP-associated modulation of host antiviral responses. Importantly, the physicochemical mechanisms proposed to underlie PS-MP-associated effects on enveloped viruses—including hydrophobic and electrostatic interactions and PS-MP-induced alterations in host cell membrane properties—are inferred from the existing literature and were not directly validated in the present study. The absence of direct experimental evidence, such as virus–PS-MP binding assays, coprecipitation analyses, or ultrastructural imaging, therefore represents a key limitation. In addition, pristine polystyrene microplastics were used in this study, whereas environmental microplastics are typically subjected to aging processes, such as UV irradiation, mechanical abrasion, and chemical weathering. These processes can induce physicochemical alterations, including increased surface roughness, surface oxidation, and changes in zeta potential,^2,30^ which may substantially influence particle–virus interactions and adsorption behavior. As a result, virus–microplastic interactions observed using pristine particles may not fully capture the complexity of environmentally aged microplastics. Furthermore, intracellular PS-MP uptake was not quantified, potentially limiting the interpretation of the relative contributions of extracellular versus intracellular interactions. If PS-MPs are predominantly localized extracellularly, the observed effects are consistent with proposed mechanisms involving viral shielding, enhanced viral stability, and alterations in host cell membrane properties. Conversely, intracellular uptake may contribute to the stress-mediated modulation of antiviral signaling. Future studies incorporating protein-based analyses, direct virus–microplastic interaction assays, environmentally aged particles, and uptake quantification will be important to further refine the mechanistic understanding and environmental relevance.

## Conclusions

This study demonstrates that PS-MPs can influence host–virus interactions by inducing cellular stress and suppressing innate antiviral immune responses. PS-MP exposure was associated with increased detectable infection levels and enhanced low-temperature persistence of enveloped viruses (HCoV-OC43 and RSV), whereas no comparable effects were observed for the nonenveloped virus EV-A71. These findings underscore the importance of viral structural features in shaping microplastic-associated viral outcomes. Overall, the results suggest that microplastics may function as environmental cofactors that modulate viral persistence and host susceptibility, highlighting the need to consider microplastic pollution in the context of viral ecology and public health risk.

## Supplementary Material



## Data Availability

All data supporting the findings of this study are included in the main text and the Supporting Information.

## Notes

Figure S1, Viral persistence at room temperature (30 °C). HCoV-OC43, RSV, and EV-A71 were incubated with or without PS-MPs at 30 °C, and viral survival was assessed on days 3, 5, and 7 by plaque assay. No infectious virus was detected above the assay limit of detection at any time point or condition. Table S1, Physicochemical characteristics of polystyrene microplastics (PS-MPs) used in this study. Table S2, Comparison of PS-MP concentrations used in this study with reported environmental and human exposure levels (PDF)
