# Supplementary material for: Polystyrene Microplastic-Induced Cellular Alterations and Their Effects on Viral Entry (RSV, HCoV-OC43, EV-A71) and Viral Persistence
Source: Environ Health (Wash). 2026 Feb 5;4(5):1040–9. doi: 10.1021/envhealth.5c00613 (PMC13185058; doi:10.1021/envhealth.5c00613)
Supplement: Supplementary file 1 [file eh5c00613_si_001.pdf]

Supporting Information

Polystyrene Microplastic-Induced Cellular Alterations and Their Effects on Viral Entry (RSV, HCoV-OC43, EV-A71) and Viral Persistence

Nattamon Niyomdecha[1*], Pornprapa Srimorkun[2], Jarunee Prasertsopon[3], and Kittisak Suanpan[4]

[1] *Department of Medical Technology, Faculty of Allied Health Sciences, Thammasat University, Rangsit Campus, Pathum Thani, Thailand*

[2] *Graduate Program in Biomedical Sciences, Faculty of Allied Health Sciences, Thammasat University, Pathum Thani, Thailand*

[3] *Center for Research Innovation and Biomedical Informatics, Faculty of Medical Technology, Mahidol University, Nakhon Pathom, Thailand*

[4] *Department of Pediatrics, Faculty of Medicine Ramathibodi Hospital, Mahidol University, Bangkok, Thailand*

[*]**Corresponding author:** Assoc. Prof. Nattamon Niyomdecha, Ph.D.
Department of Medical Technology,
Faculty of Allied Health Sciences, Thammasat University
E-mail: nattamon@tu.ac.th (+66 86-838-9005)
ORCID ID: https://orcid.org/0000-0002-5364-6716

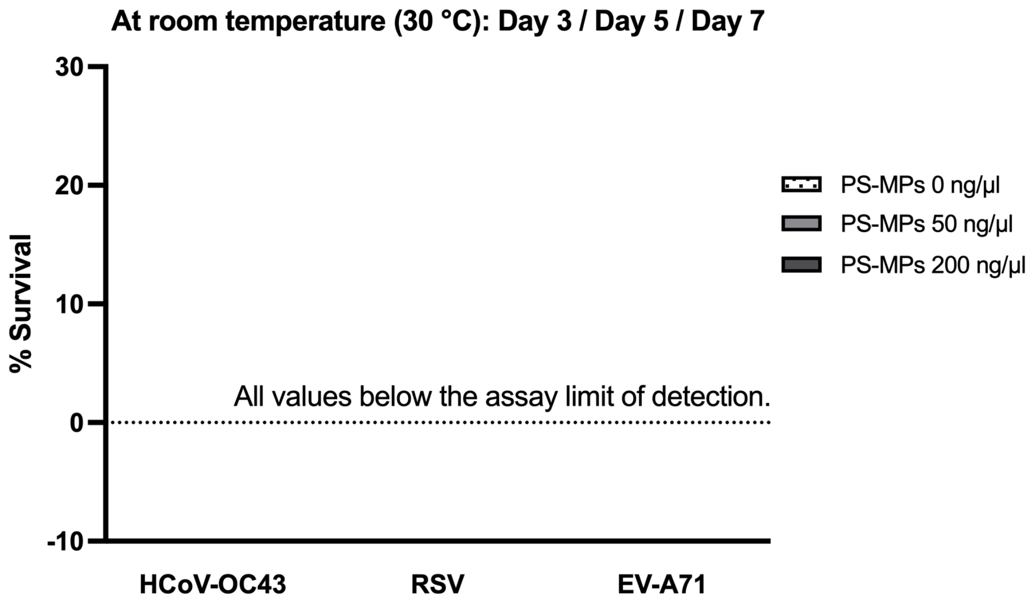

Figure S1. Viral persistence at room temperature (30 °C). HCoV-OC43, RSV, and EV-A71 were incubated with or without PS-MPs at 30 °C, and viral survival was assessed at days 3, 5, and 7 by plaque assay. No infectious virus was detected above the assay limit of detection at any time point or condition.

Table S1. Physicochemical characteristics of polystyrene microplastics (PS-MPs) used in this study

Parameter	Specification	Description / Notes
Product name	Polystyrene micro particles	Commercial latex bead formulation
Manufacturer	Sigma-Aldrich (Merck KGaA)	MilliporeSigma
Catalog number	43302	Lot-specific certificate of analysis provided
Particle composition	Polystyrene (PS)	Non-functionalized polymer
Nominal particle diameter	100 nm	As specified by manufacturer
Calibrated mean diameter	0.113 µm (113 nm)	Determined by CPS disc centrifuge
Particle size distribution (SD)	0.003 µm (3 nm)	Narrow distribution
Coefficient of variation (CV)	2.8%	Indicates high size uniformity
Measurement technique	CPS Disc Centrifuge (DC-24000)	NIST-traceable calibration
Particle morphology	Spherical latex beads	Typical PS microsphere morphology
Density	1.05 g/cm³	Particle specific gravity
Physical state	Aqueous suspension	Ready-to-use stock
Solid content	10% (w/v)	Stock concentration
Water dispersibility	Freely dispersible	Suitable for cell-based assays
Surface functionalization	None	Plain PS particles
Zeta potential	Not specified by supplier	Typically mildly negative for non-functionalized PS
Storage condition	2–8 °C	As recommended by manufacturer
Chemical stability	Stable under standard laboratory conditions	No known degradation during storage

Note: Particle size and distribution were determined by the manufacturer using CPS disc centrifuge analysis with NIST-traceable standards. Zeta potential values were not provided in the certificate of analysis; however, non-functionalized polystyrene latex particles are commonly reported to exhibit a mildly negative surface charge in aqueous systems, which may influence nanoparticle–virus and nanoparticle–cell interactions.

Table S2. Comparison of PS-MP concentrations used in this study with reported environmental and human exposure levels

Matrix / Microenvironment	Reported microplastic concentration	Unit	Particle type / size (if available)	Detection method / Study type	Reference
Surface water / Drinking water	~10^2–10^5	particles/	Mixed polymers (<5 mm)	Filtration + μFTIR / Raman	Koelmans et al., 2019 [2]
Indoor / outdoor air	~0.3–9.6	particles/m³	Fibers & fragments (<100 μm)	Air sampling + μFTIR	Zheng et al., 2024 [3]
Human sputum (respiratory samples)	~0.1–10	particles/sample	PS, PE (<50 μm)	Digestion + μFTIR	Huang et al., 2022 [5]
Human blood	~1–10	particles/ml	PS, PMMA (7–30 μm)	Py-GC/MS	Leslie et al., 2022 [4]
Human placenta / tissue	~0.3–1.6	particles/g tissue	PS, PP (5–10 μm)	Raman microscopy	Ragusa et al., 2021 [6]
This study (*in vitro* exposure)	25–1000	ng/μl	PS, ~100 nm	Controlled cell-culture exposure	This study

Note: Environmental and human exposure levels are typically reported as bulk concentrations or particle counts. In contrast, the concentrations applied in this study represent localized, controlled exposures at the cell–particle interface and were used as a mechanistic screening range rather than a direct simulation of ambient environmental levels.

Abbreviations: MPs, microplastics; PS, polystyrene; PE, polyethylene; PP, polypropylene; PMMA, polymethyl methacrylate; μFTIR, micro–Fourier transform infrared spectroscopy; Py-GC/MS, pyrolysis–gas chromatography/mass spectrometry. **Units:** μm, micrometer; ng/μl, nanograms per microliter.
